# *APOE* Genotype and Endothelial Biomarkers: Towards Personalized Cardiovascular Screening

**DOI:** 10.3390/genes16121494

**Published:** 2025-12-15

**Authors:** Gisella Titolo, Mariarosaria Morello, Silvia Caiazza, Ettore Luisi, Achille Solimene, Chiara Serpico, Saverio D’Elia, Paolo Golino, Francesco S. Loffredo, Francesco Natale, Giovanni Cimmino

**Affiliations:** 1 Department of Advanced Medical and Surgical Sciences, University of Campania Luigi Vanvitelli, Piazza, Luigi Miraglia, 2, 80138 Naples, Italy; gisellatitolo@gmail.com (G.T.);; 2 Department of Translational Medical Sciences, Section of Cardiology, University of Campania Luigi Vanvitelli, 80131 Naples, Italy; 3 Cardiology Unit, Azienda Ospedaliera Universitaria Luigi Vanvitelli, 80138 Naples, Italy; 4 Vanvitelli Cardiology and Intensive Care Unit, Monaldi Hospital, 80131 Naples, Italy; 5 Department of Life Science, Health, and Health Professions, Link Campus University, 00165 Rome, Italy

**Keywords:** Apolipoprotein E, ApoE genotype, cardiovascular risk, endothelial dysfunction, thrombosis

## Abstract

Cardiovascular diseases represent one of the leading causes of morbidity and mortality worldwide despite tremendous advancements in therapeutic interventions. Prevention remains one of the most effective strategies to reduce individual risk. Apolipoprotein E (ApoE), through its genetic variants (*ε*2, *ε*3, *ε*4), is a well-known modulator of cardiovascular risk, traditionally studied for its role in lipid metabolism. However, recent evidence suggests that ApoE also influences endothelial function and thrombotic processes, opening new perspectives for an integrated approach to risk assessment. This narrative review explores the potential of using the *APOE* genotype as a key genetic biomarker, integrated with emerging endothelial markers (e.g., plasma levels of endothelin-1, nitric oxide, von Willebrand factor, endothelial adhesion molecules) to achieve a more accurate and personalized stratification of cardiovascular and thrombotic risk. The combined approach may overcome the limitations of traditional thrombophilia screening, which is often poorly informative when performed without clear clinical criteria, and may guide more targeted therapeutic decisions, particularly in borderline-risk individuals or those with unexplained thrombotic events. Finally, the review discusses the clinical implications, current challenges, and future perspectives for integrating this model into clinical practice within the framework of precision medicine. The early identification of genetically predisposed patients, together with functional endothelial assessment, could represent a breakthrough in modern cardiovascular prevention.

## 1. Introduction

Cardiovascular (CV) and thrombotic diseases remain leading causes of illness and death worldwide, despite major advances in therapy and prevention [1]. One persistent challenge is identifying those truly at risk. Conventional thrombophilia screening often relies on standard tests [2] that, while useful in selected contexts, tend to provide little predictive value when applied broadly. Many patients at real risk remain unidentified, whereas others undergo testing that adds little to clinical decision-making. This highlights the need for more refined and individualized approaches. In recent years, the growing field of personalized medicine has started to change our perspective. Instead of applying a “one-size-fits-all” model, we now look at how genetic background and functional markers can together shape CV risk. Among genetic factors, Apolipoprotein E (ApoE) is particularly intriguing. Its isoforms (*ε*2, *ε*3, *ε*4) are well known for influencing lipid metabolism [3]. However, emerging evidence indicates that ApoE also affects endothelial biology, vascular inflammation, and thrombotic pathways [4]. Parallel to this, endothelial biomarkers—including endothelin-1, nitric oxide (NO), von Willebrand factor (vWF), and adhesion molecules—are proving to be sensitive indicators of vascular function and dysfunction [5]. This review discusses how these two dimensions, genetic and endothelial, can be combined into a more integrated model for CV risk assessment. The aim is to show how ApoE genotyping, when considered together with endothelial biomarkers, may help overcome the limits of traditional thrombophilia testing. Such an approach could improve the identification of individuals at borderline risk, guide more tailored preventive strategies, and ultimately bring us closer to a precision medicine framework in CV care.

## 2. Apolipoprotein E: Structure, Polymorphisms, and Functions

**Figure 1:**
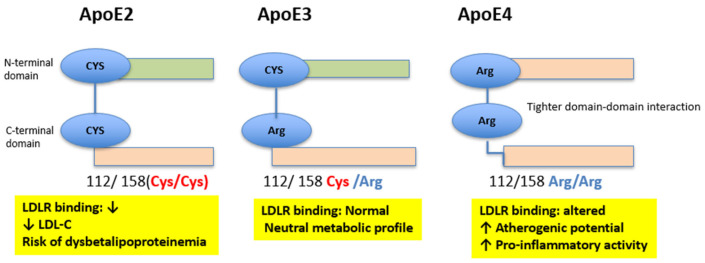
Structural differences among ApoE isoforms (ApoE2, ApoE3, and ApoE4). Schematic representation of the N-terminal and C-terminal domains of ApoE, highlighting the amino-acid substitutions at positions 112 and 158 that differentiate the three major isoforms. ApoE2 carries Cys112/Cys158, ApoE3 carries Cys112/Arg158, and ApoE4 carries Arg112/Arg158. These substitutions influence the flexibility of the helix bundle and the extent of domain–domain interaction, resulting in isoform-specific differences in LDL receptor binding capacity and metabolic phenotype.

ApoE is a 34 kDa glycoprotein encoded by the *APOE* gene on chromosome 19q13.2 [6]. It is expressed in several tissues, including the liver, brain, and vascular wall, and is essential for the clearance of triglyceride-rich lipoproteins and the maintenance of cholesterol homeostasis [7]. ApoE acts as a multifunctional ligand for several receptors involved in lipoprotein homeostasis. While ApoB100 on LDL binds primarily to the LDL receptor (LDLR), ApoE-containing lipoproteins interact with multiple receptors, including LDLR, LDL receptor-related protein 1 (LRP1), very-low-density lipoprotein receptor (VLDLR), ApoER2, and heparan sulfate proteoglycans (HSPG) [6,7,8]. This broader receptor specificity results in a more efficient clearance of triglyceride-rich lipoprotein remnants compared to LDL–ApoB mediated uptake. Through these interactions, ApoE plays a central role in the removal of chylomicron and VLDL remnants and modulates diverse metabolic and vascular processes. Three major isoforms (*ε*2, *ε*3, and *ε*4) arise from single nucleotide substitutions at codons 112 and 158, resulting in structural differences that affect receptor-binding affinity and functional properties [9]. *APOE-ε*3 is the predominant isoform and generally does not confer increased disease risk, whereas *APOE-ε*2 and *APOE-ε*4 are associated with altered lipid profiles and specific metabolic or CV outcomes [8]. The functional differences among *APOE* isoforms arise from two key amino acid substitutions at positions 112 and 158. ApoE2 contains Cys112/Cys158, ApoE3 contains Cys112/Arg158, and ApoE4 contains Arg112/Arg158. These changes alter the conformation of the N-terminal helix bundle and modulate the interaction between the N-terminal and C-terminal domains. Notably, the Arg112 residue in ApoE4 promotes tighter domain-domain interaction, reducing structural flexibility and impairing receptor-binding dynamics. These biochemical differences explain why ApoE4 exhibits reduced lipid-binding stability, increased pro-inflammatory activity, and higher atherogenic potential, while ApoE2 shows diminished LDLR affinity and predisposes to dysbetalipoproteinemia in a subset of individuals [6,8,9]. Figure 1 provides a schematic overview of these isoform-specific structural differences. 

Allelic frequencies differ across populations. In most Caucasian cohorts, ε3 accounts for approximately 70–80% of alleles, *ε*4 for 10–15%, and ε2 for 5–10% [10]. When considering genotype distribution in the general population, *ε*3*/ε*3 represents by far the most common genotype (around 60–70%), followed by heterozygous *ε*2*/ε*3 and *ε*3*/ε*4 (each occurring in approximately 10–20% of individuals). Genotypes containing *ε*4 are less frequent but clinically relevant due to their association with higher cardiovascular risk, whereas *ε*2*/ε*2 is rare (<1%) and mainly linked to dysbetalipoproteinemia under specific metabolic conditions. These patterns are confirmed in recent studies, although notable variations exist depending on ethnicity and disease status. For example, the ε4 allele is enriched in patients with coronary atherosclerosis compared with controls, suggesting a contribution to CV risk [11] whereas *ε*2 may provide partial protection in certain groups but predisposes to dysbetalipoproteinemia under specific metabolic conditions [12]. 

**Figure 2:**
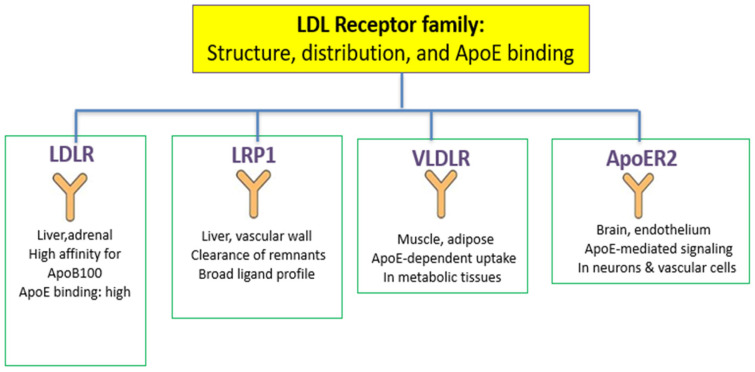
Overview of the LDL receptor family and their tissue distribution, ligand preferences, and ApoE-binding properties. The schematic view illustrates the main members of the LDL receptor family—LDLR, LRP1, VLDLR, and ApoER2—highlighting their predominant tissue expression and functional roles. LDLR displays high affinity for ApoB100 and also binds ApoE; LRP1 is central to ApoE-rich remnant clearance; VLDLR mediates ApoE-dependent uptake in metabolic tissues; and ApoER2 participates in ApoE-mediated signaling in neuronal and vascular cells.

ApoE functions as ligand for members of the LDL receptor family, facilitating lipoprotein clearance and maintaining cholesterol homeostasis [7]. The LDL receptor family comprises several structurally related endocytic receptors, including LDLR, LRP1, VLDLR and ApoER2 as shown in Figure 2. 

**Figure 3:**
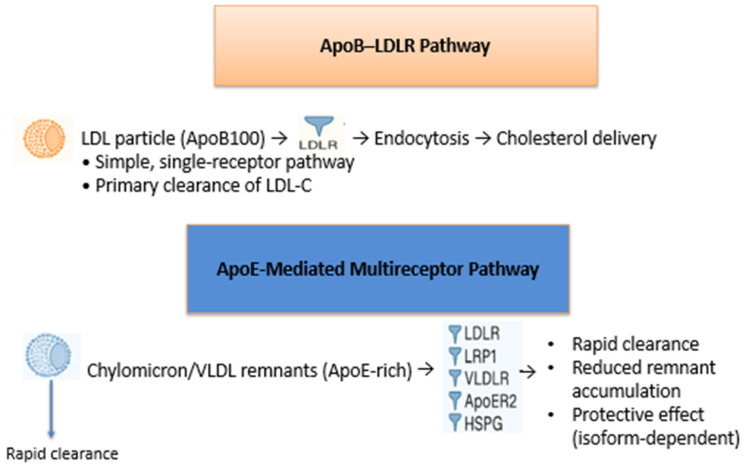
Comparison between the ApoB–LDLR clearance pathway and the ApoE-mediated multireceptor remnant uptake. The upper panel illustrates the classical LDL clearance route, in which ApoB100-containing LDL particles bind exclusively to LDLR, leading to internalization and cholesterol delivery through a single-receptor system. The lower panel depicts the ApoE-dependent pathway, where ApoE-rich chylomicron and VLDL remnants interact with multiple receptors—including LDLR, LRP1, VLDLR, ApoER2 and HSPG—resulting in faster clearance, reduced remnant accumulation, and isoform-dependent protective effects.

Although they share common ligand-binding repeats and internalization motifs, they differ in tissue distribution and preferential ligand affinity. LDLR primarily mediates the uptake of LDL particles via ApoB100, but it also binds ApoE with high affinity. LRP1 is highly expressed in the liver and vascular wall and plays a major role in the clearance of ApoE-rich remnants. VLDLR and ApoER2 are expressed in metabolic and vascular tissues and contribute to ApoE-dependent lipoprotein internalization. Together, these receptors ensure efficient removal of remnants and maintain cholesterol balance. Figure 2 summarizes the structural and functional differences among LDLR, LRP1, VLDLR, and ApoER2. A schematic comparison of the ApoB–LDLR pathway and the ApoE-mediated multireceptor clearance route is shown in Figure 3. 

Evidence from transgenic and knockout mouse models has highlighted the isoform-specific effects of ApoE, showing how deficiency accelerates atherosclerosis and how human-like isoform knock-in reproduces distinct lipid and vascular phenotypes [13]. Beyond lipid metabolism, ApoE also modulates endothelial function, inflammation, and oxidative stress. Isoforms differ in their effects on vascular biology. ApoE can regulate immune responses through the NLRP3 inflammasome, linking genotype to systemic inflammatory status [14]. In the vascular system, ApoE isoforms influence endothelial integrity and leukocyte adhesion, with ε4 often associated with impaired vascular homeostasis [15]. Recent work in brain pericytes shows that ApoE modulates endothelial function in an isoform-dependent manner by altering basement membrane composition, thereby affecting vascular stability [16]. These findings suggest that isoform-specific ApoE effects on pericyte-endothelial interactions may have broader relevance for systemic vascular beds. Taken together, these data indicate that ApoE functions extend beyond lipid transport, influencing vascular biology, immunity, and oxidative stress, and representing a key genetic determinant of CV risk [17]. These structural and receptor-binding differences highlight that ApoE isoforms do not merely influence lipid metabolism but also modulate endothelial signaling, oxidative stress responses, and vascular inflammation in an isoform-specific manner. The revised mechanistic framework presented in this section, supported by the new diagrams, provides a clearer understanding of how ApoE genotype contributes to inter-individual variability in cardiovascular and thrombotic risk.

## 3. ApoE in the Cardiovascular Scenario Stratification

Numerous studies have investigated the role of ApoE polymorphisms in coronary artery disease (CAD) [18]; in particular, ApoE-ε4 allele has been extensively studied for its role in CV and cerebrovascular (CBV) diseases. Epidemiological and genetic evidence consistently demonstrates that ApoEε4 allele is a significant risk factor for CAD. Multiple meta-analyses and large-scale studies show that individuals carrying the ε4 allele, particularly in the ε3/ε4 or ε4/ε4 genotypes, have an increased risk of CAD compared to those with the ε3/ε3 genotype, with odds ratios often around 1.5–2.0, independent of traditional risk factors and across diverse populations [19,20,21,22,23,24]. The ε4 allele is associated with higher plasma levels of low-density lipoprotein cholesterol (LDL-C) and total cholesterol, which partly explains its atherogenic potential [21,25,26]. Notably, the ε4-related risk may be more pronounced in older individuals and in those with comorbidities such as type 2 diabetes mellitus (DM) [22,23,25].

Furthermore, regarding ischemic stroke, case–control and meta-analytic studies indicate a significantly higher ApoE-ε4 allelic frequency in affected patients compared with control subjects, with odds ratios typically ranging from 1.4 to 1.7 [24,27,28]. The association is particularly strong for large artery atherosclerosis subtypes, and ε4 carriers often exhibit higher LDL-C and total cholesterol levels, further contributing to stroke risk [24,28]. Additionally, low ApoE plasma concentrations, which may reflect the ε4 genotype, are predictive of recurrent CBV events [29].

In the context of atherosclerosis, the ε4 allele is linked to increased carotid intima-media thickness, greater atheroma burden, and a higher prevalence of carotid and coronary plaques [30,31,32]. Meta-analyses confirm that ε4 homozygosity significantly increases the risk of carotid atherosclerotic plaque, and the presence of at least one ε4 allele is associated with a higher risk of subclinical and clinical atherosclerosis in multiple vascular beds [30,31,33,34]. The atherogenic effect of ε4 is mediated by its impact on lipid metabolism and pro-inflammatory pathways, as supported by omics analyses [33].

The clustering of multiple lifestyle risk factors—physical inactivity, poor diet, smoking, and hypertension—has a particularly strong adverse effect among ε4 carriers, showing then the importance of comprehensive lifestyle modification in this group. Smoking interacts with ApoE genotype to amplify CV risk, particularly among ε4 carriers, who experience a greater increase in coronary heart disease (CHD) and mortality compared to non-carriers; this effect is especially pronounced in women and current smokers, but risk decreases with smoking cessation [35,36,37]. Diet also modifies ApoE-related risk: high saturated fat intake and low polyunsaturated fat intake further elevate the risk of dementia and CV disease in ε4 carriers, suggesting that dietary interventions may be particularly beneficial for genetically susceptible individuals [38,39,40]. Hypertension and dyslipidemia act synergistically with ApoE variants, increasing the risk of CAD, especially when combined with other risk factors such as smoking [41,42,43]. In reality, recent metabolomic and lipidomic studies have revealed that ApoE polymorphisms, particularly the ε4 allele, are linked to a wide range of metabolic alterations including shifts in glycoprotein acetyls, LDL particle size, isoleucine levels, and other emerging biomarkers, highlighting the need to move beyond standard lipid profiles and explore a broader set of biomarkers to fully capture the complexity of metabolic health [44,45,46].

## 4. ApoE and Endothelial Function

ApoE is primarily known for its role in lipid metabolism and atherosclerosis susceptibility, but it also exerts significant effects on the vascular endothelium. ApoE influences endothelial nitric oxide synthase (eNOS) activity, NO bioavailability, expression of adhesion molecules, and oxidative stress balance, thereby impacting vascular homeostasis [47,48].

ApoE modulates endothelial NO production through mechanisms involving caveolin-1 and eNOS interactions. Yue et al. demonstrated that ApoE enhances endothelial NO generation by modulating the caveolin-1/eNOS complex, increasing vasodilatory capacity in vitro and in vivo [47]. This interaction suggests that ApoE supports vascular function by sustaining eNOS activity and preventing its inhibition. Conversely, experimental models of ApoE deficiency show impaired endothelium-dependent relaxation, emphasizing ApoE essential role in maintaining NO-mediated vascular homeostasis [49]. ApoE deficiency promotes endothelial activation, marked by the upregulation of vascular cell adhesion molecule-1 (*VCAM-1*) and intercellular adhesion molecule-1 (*ICAM-1*). Nakashima et al. showed that these adhesion molecules are markedly increased in atherosclerosis-prone vascular sites in ApoE-deficient mice, facilitating leukocyte adhesion and early lesion formation [50]. Such endothelial activation contributes to inflammation and accelerates atherogenesis. This aspect was also observed in endothelial cells (ECs), transfected in vitro to secrete ApoE, with reduction in VCAM-1 expression [51]. Furthermore, ApoE can reduce CD4 and CD8 T cells proliferation, determining also a reduced expression of major histocompatibility complex class II molecules which reflects in a minor T cells antigen mediated activation. It is also implied in innate immune response because tumor necrosis factor (TNF) increases ApoE expression, while interferon gamma decreases its expression. ApoE stimulates synthesis of macrophages M2 markers (as arginin-1) which, in contrast to M1 type, determine a reduction in atherosclerosis and inflammation [52].

ApoE deficiency is also associated with oxidative stress and redox imbalance in vascular tissues. Indeed, another study demonstrated that ApoE-knockout (ApoE−/−) induces vascular oxidative stress, leading to redox gene dysregulation, increased reactive oxygen species (ROS) formation and activation of inflammatory pathway [48]. This oxidative environment further reduces NO bioavailability and enhances endothelial dysfunction.

The ApoE-knockout mouse represents the standard experimental model for studying atherosclerosis and endothelial dysfunction. These mice exhibit impaired endothelium-dependent vasorelaxation and increased expression of adhesion molecules, oxidative stress, and vascular inflammation compared with controls [48,50]. Overexpression of eNOS in ApoE-deficient mice, however, has been shown to accelerate lesion formation, suggesting that unbalanced NO production can also be detrimental [49]. Human studies are still limited but it is observed a reduced CV risk in patient with ε2 allele expression [53].

## 5. Endothelial Biomarkers: State of the Art

ECs have an important role in maintaining vascular homeostasis through the regulation of vascular tone, permeability, and inflammation. Circulating or locally released endothelial biomarkers provide a dynamic view of vascular function and injury, and their inclusion into clinical research has improved CV and thrombotic risk stratification.

NO is generated by eNOS. It represents the primary mediator of vasodilation and it is an essential regulator of vascular integrity. By activating soluble guanylate cyclase and increasing cyclic GMP, NO promotes vasodilation, inhibits platelet aggregation, and reduces leukocyte adhesion. Impaired eNOS activity or oxidative inactivation of NO contribute to endothelial dysfunction in atherosclerosis [54].

Endothelin-1 (ET-1), a 21-amino acid peptide produced by ECs, is one of the most potent endogenous vasoconstrictors. Elevated ET-1 expression determines vascular smooth muscle proliferation, oxidative stress, and fibrosis. Furthermore, chronic ET-1 elevation is responsible for vascular remodeling and microvascular complications in hypertension and heart failure (HF) [55].

vWF, a multimeric glycoprotein stored in Weibel–Palade bodies of ECs and in platelet α-granules, is rapidly released into circulation or exposed on the vascular surface upon endothelial activation/injury. vWF links endothelial activation to thrombosis by mediating platelet adhesion under high arterial shear stress. Elevated plasma vWF antigen or activity reflects endothelial damage and is associated with thrombotic risk. Clinical studies have linked high vWF levels with ischemic stroke, venous thromboembolism (VTE), and adverse outcomes in inflammatory and infectious conditions including COVID-19 [56,57,58,59].

Adhesion molecules (ICAM-1 and VCAM-1) are upregulated on the endothelial surface in response to inflammatory cytokines such as Tumor necrosis factor-α (TNF-α) and Interleukin-6 (IL-6), and induce leukocyte migration during inflammation. Their key role in regulating homeostasis and in CVD such as atherosclerosis, atrial fibrillation (AF), myocardial infarction (MI) and stroke is established [60].

TNF-α is a pleiotropic pro-inflammatory cytokine that disrupts endothelial function by increasing oxidative stress, impairing eNOS phosphorylation, and promoting adhesion molecule expression. IL-6 is a central mediator of vascular inflammation and a key downstream effector of innate immunity. It promotes hepatic synthesis of acute-phase reactants (including fibrinogen and C-reactive protein), enhances platelet production and activation, and directly contributes to endothelial oxidative stress and impaired NO signaling. Elevated levels of IL-6 are associated with a higher risk of CV death, major adverse cardiovascular events, MI, stroke, peripheral artery disease (PAD), and HF. A recent study shows that combined TNF-α and IL-6 signaling drives oxidative stress, suppresses eNOS, and blunts coronary microvascular vasodilation; conversely, blocking either cytokine improves endothelial function in diabetic models [61,62].

The integration of endothelial activation markers with host genetics may improve CV risk stratification. Among genetic modulators, ApoE isoforms play a fundamental role in lipoprotein metabolism, oxidative stress, and vascular inflammation. ApoE loss-of-function (as in ApoE-knockout mice) accelerates endothelial oxidative stress, increases leukocyte adhesion, and worsens atherosclerotic plaque burden [48]. ApoE polymorphisms influence plasma lipid profiles and endothelial function and are linked to atherosclerotic CV risk [53]. Thus, combining dynamic endothelial biomarkers (NO, ET-1, vWF, ICAM-1/VCAM-1, IL-6, TNF-α) reported in Table 1, with stable genetic factors such as ApoE genotype could improve early identification of high-risk patients and guide targeted preventive strategies. 

## 6. Integration of ApoE and Endothelial Biomarkers in Clinical Screening

### 6.1. Indications for ApoE Genotyping Testing

Designing a personalized screening model that integrates ApoE genotyping with endothelial biomarkers requires a shift from a static, population-based view of risk assessment to a dynamic, biologically informed context. Rather than expanding the list of tests, the aim is to propose a sequential strategy that couples a stable genetic background marker with a set of functional data reflecting vascular health. ApoE genotyping should not be considered a universal screening tool, but rather a stratification instrument applied selectively where it may refine clinical decision-making [63].

Among six common ApoE genotypes (three homozygous: ε2/ε2, ε3/ε3, ε4/ε4; three heterozygous: ε2/ε3, ε3/ε4, ε2/ε4), those carrying at least one ε4 allele (i.e., ε2/ε4, ε3/ε4, and especially ε4/ε4) exhibit a consistently higher risk of CAD due to the adverse effects of ε4 on lipid regulation. It is linked to elevated levels of LDL-C, total cholesterol, and VLDL, likely through downregulation of LDL receptor (LDLR) expression, leading to reduced lipid clearance [64,65,66,67]. Carriers of the ε4/ε4 genotype have the highest LDL-C levels and a significantly higher risk for CAD [68], though not for stroke [69]. The association between ε4 and CAD risk is especially relevant in certain populations, such as Caucasians and Asians [65,66]. In contrast, genotypes carrying the ε2 allele (ε2/ε2 and ε2/ε3) are generally considered protective, associated with lower LDL-C but higher triglycerides and non-HDL-C [68,70]. Although ε2/ε2 individuals typically have a lipid profile protective against CAD, around 15% develop familial dysbetalipoproteinemia (FD), a disorder linked to increased risk of PAD [71]. In FD patients, PAD prevalence has been reported as 6.5% and 11% in different cohorts, compared to 0.3% in healthy controls [72,73]. While ApoE has been associated with CHD and stroke, its relationship with PAD is less clear. A study regarding elderly Japanese-American men found no significant link between ApoE genotype and PAD, but only a limited range of genotypes were examined [74]. However, a lower frequency of ε2-containing genotypes among patients undergoing carotid endarterectomy compared to controls suggests a protective role of ε2 in carotid atherosclerosis [75].

Moreover, *APOE* genotyping may be particularly informative in patients with thrombotic events despite negative results on conventional thrombophilia panels. Evidence indicates that individuals carrying ε4-containing genotypes (ε2/ε4, ε3/ε4, ε4/ε4) exhibit a pro-inflammatory and pro-oxidative vascular phenotype, characterized by endothelial activation, impaired nitric oxide bioavailability, and increased expression of adhesion molecules, all of which contribute to a prothrombotic milieu [63,76].

Finally, ApoE genotype testing is particularly useful in individuals with intermediate or unclear CV risk and early-onset CAD, hyperlipidemia, or a family history of premature events [77,78,79]. Combined with endothelial biomarkers, it enables personalized risk stratification and targeted preventive strategies, avoiding universal screening.

#### 6.1.1. Targeting Endothelial Biomarkers: Which Patients Benefit Most?

Endothelial biomarkers, such as soluble adhesion molecules, including sICAM-1, sVCAM-1, and sE-selectin—as well as endothelial microparticles (EMPs), have become increasingly important in the assessment of endothelial activation, vascular inflammation, and the progression of atherosclerosis [80]. In individuals with carotid atherosclerosis or subclinical CHD, soluble adhesion molecules like sE-selectin and ICAM-1 can reveal endothelial activation before clinical symptoms appear, allowing timely preventive measures [81]. PAD patients benefit from monitoring sICAM-1, IL-6, and CRP, which reflect ongoing vascular inflammation and predict disease progression, particularly in the lower limbs [82]. For those experiencing acute coronary syndromes, including MI and unstable angina, EMP levels are elevated and correlate with systemic inflammation markers such as IL-6 and CRP, providing insight into acute endothelial injury and thrombosis risk [83]. EMPs are also informative in stable and unstable angina, helping to differentiate the degree of endothelial activation and guide treatment decisions. Other high-risk populations include patients with DM and severe hypertension, in whom EMP monitoring can signal early vascular complications and ongoing endothelial injury. Similarly, individuals with immune-mediated vascular diseases including microscopic polyangiitis, polyarteritis nodosa, and Takayasu arteritis, benefit from EMP assessment to monitor disease activity and response to therapy [84,85]. Overall, endothelial biomarkers provide a promising approach to understanding vascular health across a broad range of conditions, enabling earlier detection, better risk assessment, and more personalized patient care.

Table 2 summarizes interaction between ApoE genotype and endothelial biomarkers as part of the precision medicine perspective.

#### 6.1.2. How Can Biomarker Data Be Integrated with Traditional Thrombophilia Screening?

Integrating genetic data into traditional thrombophilia screening may provide new insights into thrombotic susceptibility. Recent evidence suggests that ApoE polymorphisms may influence susceptibility to deep vein thrombosis (DVT). In a case–control study, Zhu et al. analyzed 300 DVT patients and 300 matched healthy controls, reporting that individuals with the ApoE ε3/ε4 genotype had a moderately increased DVT risk [87]. Nagato et al. corroborated these findings in a smaller cohort, observing a higher prevalence of the ε2 allele among female patients compared with controls (11% vs. 0%), suggesting a potential sex-specific influence [88]. To explain this association, Ulrich et al. proposed a model in which ApoE isoforms differentially affect endothelial function via ApoER2 signaling. ApoE ε3, the wild-type variant, exerts protective effects by promoting eNOS activation, NO production, EC migration, and reduced leukocyte adhesion—mechanisms that collectively support vascular health [89]. In contrast, ApoE ε4 displays pro-thrombotic and pro-inflammatory features, potentially exacerbated by antiphospholipid antibodies that impair ApoER2 signaling, thereby amplifying thrombotic risk [90]. Although some studies, such as those conducted in North Indian cohorts, did not find statistically significant allele differences, the overall evidence suggests that ApoE polymorphisms may interact with other thrombophilic factors, modulating thrombotic susceptibility [91].

Evaluating ApoE genotype, particularly distinguishing ApoE ε3 from ApoE ε4 carriers, could therefore add value to traditional thrombophilia screening, especially in complex or unexplained thrombotic cases. The endothelial-protective role of ApoE3 supports its potential use as a complementary genetic marker, aiding in more precise patient stratification and personalized prophylactic or therapeutic strategies.

### 6.2. Expected Benefits

#### 6.2.1. Improved Accuracy in Risk Stratification

ApoE genotyping is emerging as a valuable tool for enhancing cardiovascular risk stratification, especially when integrated with clinical variables and endothelial biomarkers. Traditionally, ApoE has been studied for its effect on LDL-C; however, recent research has expanded its relevance to other lipid-related markers such as HDL-C, triglycerides, and lipoprotein particle size. Notably, these effects are sex-specific, with studies—such as that by Nagato et al. demonstrating that the ε2 allele exerts more pronounced protective effects in women compared to men [88,92,93].

Beyond lipid metabolism, ApoE genotype appears to modulate systemic inflammation, a key driver of vascular injury. A consistent gradient in circulating CRP levels has been observed across genotypes (ε2 > ε3 > ε4), reinforcing the connection between ApoE and inflammatory risk profiles [94]. This inflammatory modulation is clinically relevant, as elevated CRP is a known predictor of CV events.

Importantly, interactions between ApoE and modifiable factors like body weight further refine individual risk stratification. Kofler et al. demonstrated that ε2 carriers are more susceptible to the metabolic consequences of obesity, showing marked increases in triglycerides, CRP, and P-selectin—an endothelial activation marker—at higher BMI levels. Conversely, ε3 and ε4 carriers appear less sensitive to these changes. This suggests that the cardioprotective role of the ε2 allele is context-dependent, potentially lost in the presence of obesity [95].

**Figure 4:**
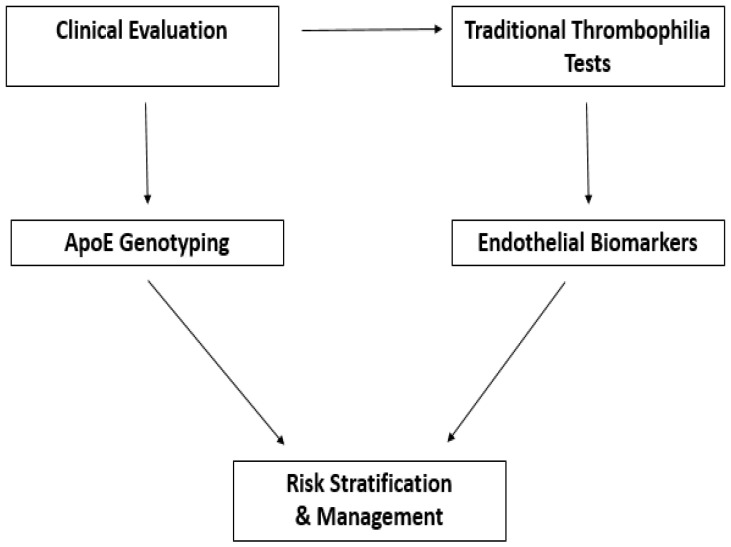
Thrombotic risk assessment integrating *APOE* genotyping and endothelial biomarkers. Initial clinical evaluation guides both traditional thrombophilia testing and *APOE* genotyping. Parallel assessment of endothelial biomarkers provides complementary information on vascular function. Data from these evaluations converge to stratify thrombotic risk and guide personalized patient management. The diagram highlights the points in the diagnostic workflow where ApoE may influence decision-making and risk assessment.

Taken together, these findings highlight the added predictive value of combining *APOE* genotyping with endothelial and inflammatory biomarkers, particularly when considered alongside physiological modifiers such as sex and BMI as reported in Figure 4.

This integrative approach moves beyond traditional risk factor models, offering a more precise and individualized framework for assessing CHD risk and guiding preventive strategies.

#### 6.2.2. Targeted Patient Selection for Treatment or Monitoring

Although *APOE* genotyping is not currently recommended for routine clinical use, emerging evidence suggests it may be valuable in guiding targeted interventions for selected patient populations. Rather than acting as a direct cause, *APOE*—particularly the ε4 allele—modulates disease risk through its interaction with environmental and metabolic factors. This opens the door to its use in identifying individuals who may benefit from personalized monitoring or preventive strategies.

One such group includes *APOE* ε4 carriers with unhealthy lifestyle factors—such as obesity, smoking, or high alcohol consumption—may be at increased risk of vascular dysfunction and adverse lipid changes [96,97]. These individuals show greater elevations in triglycerides, β-lipoproteins, insulin, and LDL-C compared to non-carriers [98,99]. Therefore, patients with these risk factors could benefit from *APOE* genotyping to allow for more personalized prevention strategies, particularly for CVD.

Another important subgroups includes individuals with a family history of type III dysbetalipoproteinemia—a rare lipid disorder associated with *APOE*2 homozygosity (*APOE* ε2/ε2)—are strong candidates for ApoE genotyping [100]. Although only a small proportion of *APOE* ε2/ε2 individuals develop the disease, early identification can enable monitoring and control of secondary risk factors, potentially delaying or preventing its onset.

Finally, in patients with type 2 DM or dyslipidemia, *APOE* genotyping may guide dietary and exercise-based interventions. For example, *APOE* ε4 carriers respond more robustly to reduced-fat diets with significant reductions in LDL-C and total cholesterol [101], and may also experience greater HDL increases from physical activity [102,103], though the effect of diet on HDL remains inconclusive [104]. These findings support the notion that *APOE* screening, when applied selectively, could enhance clinical decision-making by identifying those most likely to benefit from early intervention, targeted monitoring, or lifestyle modifications as reported in Table 3.

#### 6.2.3. Reduction in Low-Yield Tests

One of the principal advantages of integrating *APOE* genotyping and endothelial biomarker evaluation into a screening framework lies in the potential to reduce the over-utilization of non-informative thrombophilia testing. Conventional thrombophilia panels are frequently used in contexts where the results do not meaningfully influence clinical management. For instance, in a retrospective study of 200 hospitalized patients, 83.8% of thrombophilia tests were deemed inappropriate according to established criteria, with most being performed during acute thrombosis or while anticoagulation therapy was ongoing [105]. The British Society for Haematology advises against indiscriminate testing, noting that routine assessment in provoked or low-risk venous thromboembolism offers minimal clinical value and contributes to unnecessary costs and increased patient anxiety. Selective biological-based testing, based on genetic and endothelial markers, could therefore improve diagnostic efficiency, reduce redundant investigations, and focus clinical resources on those most likely to benefit from prevention or targeted therapy [106].

**Figure 5:**
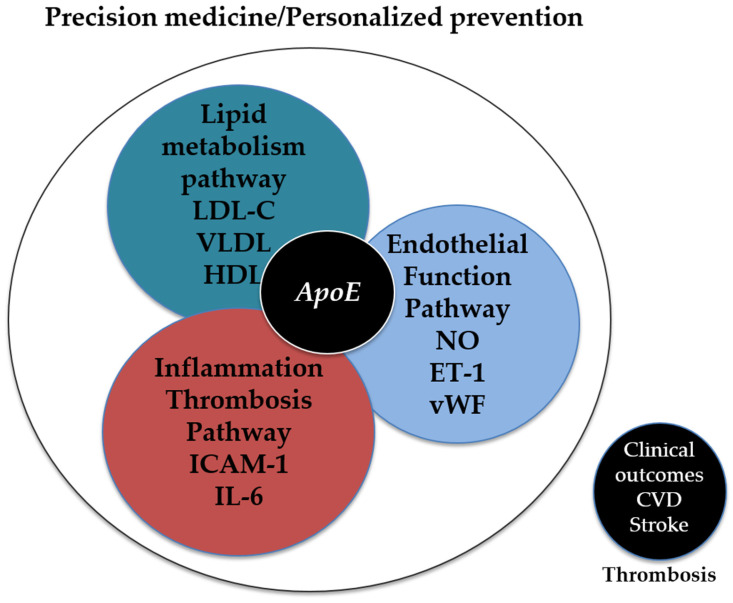
Integration of *APOE* Genotype and Pathway Interactions in Precision Cardiovascular Prevention. The figure illustrates how the *APOE* genotype acts as a central node interacting with three key pathways involved in cardiovascular risk: (1) the lipid metabolism pathway (LDL-C, VLDL, HDL), which regulates lipoprotein metabolism; (2) the inflammation and thrombosis pathway (ICAM-1, IL-6), implicated in inflammatory and thrombotic processes; and (3) the endothelial function pathway (NO, ET-1, vWF), which reflects endothelial status and vascular function. The interaction of ApoE with these pathways contributes to modulating clinical cardiovascular outcomes, including cardiovascular disease, stroke, and thrombosis. This representation highlights how integrating genetic and biological information can support tailored prevention strategies aligned with the principles of precision medicine.

A schematic view of ApoE in precision CV prevention is illustrated in Figure 5.

### 6.3. Current Limitations and Challenges: Costs and Standardization

Despite the growing interest in integrating *APOE* genotyping and endothelial biomarkers into clinical screening protocols, several limitations still hinder their routine application. These challenges primarily concern technical costs, lack of methodological standardization, and inconsistencies in clinical evidence. Several methods have been developed for *APOE* genotyping. The conventional PCR-Restriction Fragment Length Polymorphism (PCR-RFLP) technique is widely used [107,108], but it is time-consuming, error-prone, and involves multiple complex steps such as restriction enzyme digestion. More advanced techniques like PCR followed by sequencing or mass spectrometry provide accurate results but are costly and labor-intensive due to the need for high-end equipment and extensive manual handling [109]. Real-time PCR-based methods—such as High-Resolution Melt (HRM) [110,111], TaqMan probe assays [112], and Fluorescent Resonance Energy Transfer (FRET) [113]—have improved the speed and reliability of genotyping. However, these methods still present significant cost-related challenges, especially due to the use of multiple probes and potential issues like primer-dimer formation affecting curve interpretation. Despite these cost limitations, a newer, more efficient technique has been developed using an allele-specific PCR approach combined with TaqMan probe-monitored real-time PCR. This method allows for accurate ApoE genotyping in a single PCR step, without post-PCR handling, and can deliver results within 90 min. It is particularly suitable for high-throughput applications, offering improved accuracy and reduced labor and cost compared to traditional methods like PCR-RFLP [107,108].

To date, limited research has explored the relationship between circulating ApoE levels and CVD outcomes, such as stroke and CHD. One study observed that higher ApoE concentrations were linked to an increased risk of CVD events [114], a finding that contradicts previous animal and human research suggesting a protective role of ApoE. Another study conducted in the same population found a similar positive association between ApoE levels and stroke risk [115]. A third and larger study (*n* = 2951) also reported a rise in CVD incidence with higher ApoE levels, although this was confined to women with elevated high-density lipoprotein cholesterol (HDL-C) levels [116]. These results warrant replication for several reasons. First, two studies focused specifically on individuals aged 85 and older, limiting the applicability of findings to younger populations—especially given the age-related decline in the prevalence of the ε4/ε4 genotype, which is also associated with lower ApoE concentrations [117]. Second, all studies had small sample sizes and relatively few CVD outcomes (68, 54, and 156 cases, respectively). Third, and critically, there was no consistent approach to adjusting for cardiovascular risk factors across studies, highlighting a key issue of methodological heterogeneity and lack of standardization in analyzing ApoE-CVD associations. Lastly, early studies often overestimate effect sizes due to the so-called Proteus effect or winner’s curse, further emphasizing the need for independent validation [118]. In summary, while technological innovations are improving the feasibility of ApoE genotyping in clinical contexts, its widespread integration remains limited by high costs, variable technical approaches, and the need for stronger, standardized clinical evidence. Addressing these gaps is essential for translating ApoE and related biomarkers into reliable tools for personalized cardiovascular risk assessment.

## 7. Future Perspectives and Research Directions

### 7.1. The Need for Large-Scale Longitudinal Clinical Studies

The growing interest in the role of the *APOE* genotype in modulating cardiovascular and thrombotic risk highlights the importance of developing large-scale, multicenter longitudinal clinical studies. These studies should be designed to examine the relationship between the various *APOE* alleles (ε2, ε3, ε4), endothelial biomarkers, and the incidence of cardiovascular events over time. Current evidence mainly derives from observational or retrospective studies, often limited by small sample sizes or low population heterogeneity. It is well established, for example, that the *APOE* genotype is dose-dependently associated with LDL-C levels and carotid intima-media thickness and correlates with stroke risk, as demonstrated in meta-analyses including tens of thousands of subjects [69]. Well-designed prospective studies could validate the combined use of *APOE* genotyping and endothelial biomarkers as predictive tools, while also assessing the clinical impact of integrating these parameters into preventive strategies.

### 7.2. Integration into Precision Medicine Protocols

The adoption of *APOE* genotyping as a stable and easily measurable genetic biomarker aligns perfectly with the principles of precision medicine. However, its clinical implementation requires the development of shared guidelines, clearly defined interpretation criteria, and standardized analytical methodologies. The role of the ε4 allele as a cardiovascular risk factor not entirely explained by lipid profile alterations has been highlighted by recent reviews [119]. Combining *APOE* genotype data with plasma endothelial biomarkers (such as NO, ET-1, adhesion molecules, and vWF) may offer a more dynamic and individualized risk assessment, particularly valuable for asymptomatic patients or those with non-conventional risk factors. This integration could revolutionize the very concept of prevention, shifting the focus from a reactive to a proactive and personalized approach.

### 7.3. Potential of Artificial Intelligence and Big Data to Analyze Combined Genetic and Functional Variables

The application of artificial intelligence (AI) and big data analytics in biomedical research is opening new avenues for understanding the interactions between genotype, phenotype, and environment. Through machine learning algorithms, it is now possible to analyze complex, multidimensional datasets—including *APOE* variants, endothelial biomarkers, clinical parameters, medical history, and lifestyle factors. These tools can uncover hidden patterns or nonlinear correlations that would be difficult to identify using traditional statistical methods. Moreover, AI may support the development of personalized predictive models, improving risk stratification and providing real-time decision support in clinical settings [120].

### 7.4. Toward a Clinical Decision Algorithm Including ApoE

Based on current evidence, the development of a clinical decision algorithm integrating the *APOE* genotype with endothelial biomarkers and traditional risk factors appears increasingly feasible. Such an approach could be embedded into Clinical Decision Support Systems (CDSS) to provide tailored recommendations for screening, prevention, and treatment—especially in patients with uncertain risk profiles or unexplained thrombotic events. Experimental studies have suggested that *APOE* isoforms differ in their modulation of endothelial adhesion junctions and cytoskeletal organization, particularly in response to inflammatory stimuli (e.g., mCRP binding to CD31) [121]. The proposed algorithm should be validated in independent cohorts and dynamically updated to reflect scientific advances and clinical context. In the future, such a model could represent a key step toward a more effective, sustainable, and patient-centered precision cardiology.

## 8. Conclusions

ApoE, beyond its role in lipid metabolism, also affects endothelial function and thrombosis, paving the way for more personalized cardiovascular risk stratification. Integrating *APOE* genotype with endothelial biomarkers provides a more comprehensive and dynamic picture, particularly useful in individuals with unexplained events, family history, or unconventional risk factors. Despite reliable and cost-effective testing, ApoE genotyping remains underused due to lack of guidelines and limited clinical awareness. ε4 allele carriers show elevated LDL-C from a young age and higher cardiovascular risk in later life. Fully leveraging these biomarkers requires a cultural and operational shift, with greater interdisciplinary integration and revision of risk models, enabling more proactive and personalized cardiovascular prevention.

## Figures and Tables

**Table 1:** Endothelial biomarkers: a synthetic overview.

Biomarker	Origin/Mechanism	Physiological Role	Clinical Significance	References
**Nitric Oxide (NO)**	Generated by endothelial nitric oxide synthase (eNOS)	Vasodilation; platelet aggregation inhibition; leukocyte adhesion reduction	Impaired eNOS or oxidative NO inactivation contributes to endothelial dysfunction in atherosclerosis	[54]
**Endothelin-1 (ET-1)**	Produced by endothelial cells	Potent vasoconstrictor; promotes vascular smooth muscle proliferation, oxidative stress, fibrosis	Chronic elevation leads to vascular remodeling and microvascular complications	[55]
**von Willebrand Factor (vWF)**	Stored in Weibel–Palade bodies of endothelial cells and platelet α-granules	Platelet adhesion	Elevated levels indicate endothelial damage	[56,57,58,59]
**ICAM-1 and VCAM-1**	Upregulated by cytokines such as TNF-α and IL-6	Leukocyte migration during inflammation	Key role in endothelial activation and inflammation	[60]
**TNF-α**	Pro-inflammatory cytokine	Increases oxidative stress; impairs eNOS; increases adhesion molecule expression	Drives endothelial oxidative stress, suppresses eNOS	[61,62]
**IL-6**	Pro-inflammatory cytokine	Induces acute-phase reactants; enhances platelet production and activation	Drives endothelial oxidative stress, suppresses eNOS	[61,62]

**Table 2:** Integration of ApoE Genotype and Endothelial Biomarkers for Personalized Cardiovascular Risk Stratification.

Component	Description/Mechanisms	Clinical Implications	Key References
**ApoE** **Genotype**	ε2: ↓LDL-C, ↑TG; partial protection but risk of dysbetalipoproteinemia. ε3: neutral, metabolic balance. ε4: ↑LDL-C, ↑oxidative stress, ↑VCAM-1 expression, endothelial dysfunction.	Genetic basis for lipid and endothelial alterations; ε4 carriers have increased CAD and stroke risk, ε2 carriers partial protection.	[6,47,53]
**Endothelial** **Biomarkers**	↓NO bioavailability; ↑ET-1 vasoconstriction; fibrosis and vascular remodeling; ↑vWF, ICAM-1, VCAM-1; ↑IL-6, TNF-α; ↑oxidative stress and inflammation.	Reflect endothelial integrity; elevated levels indicate vascular dysfunction, inflammation, and thrombotic risk.	[54,55,60]
**Interaction** **ApoE–Endothelium**	ApoE deficiency → ↑ROS, ↑adhesion molecules, ↓NO signaling; ε4 allele amplifies oxidative and inflammatory endothelial responses	ε4 carriers show endothelial activation, oxidative stress, and vascular dysfunction.	[48,50]
**Clinical Integration**	Combined evaluation (ApoE genotype + endothelial biomarkers) for refined cardiovascular risk profiling	Avoids low-yield thrombophilia testing; identifies high-risk or borderline-risk patients	[63,86]
**Precision Medicine** **Perspective**	Integration of genetic and endothelial data enables individualized prevention and therapy	Personalized cardiovascular risk stratification; supports early detection and tailored management	[33,62]

‘↓’ indicates a decrease, ‘↑’ indicates an increase, and ‘→’ indicates no significant change.

**Table 3:** Targeted Patient Selection for *APOE* Genotyping for Personalized Preventive Strategies.

Patient Group	ApoE Genotype of Interest	Risk Factors/Clinical Context	Clinical Utility	References
**Individuals with unhealthy lifestyle (obesity, smoking, high alcohol intake)**	ε4 carriers (ε2/ε4, ε3/ε4, ε4/ε4)	Increased vascular dysfunction, elevated triglycerides, LDL-C, β-lipoproteins, insulin	Identify high-risk individuals who may benefit from personalized monitoring and preventive strategies	[96,97,98,99]
**Family history of type III dysbetalipoproteinemia**	ε2/ε2 (homozygous)	Rare lipid disorder; early identification allows monitoring of secondary risk factors	Enable early intervention, risk factor control, and potentially delay/prevent disease onset	[100]
**Patients with type 2 diabetes or dyslipidemia**	ε4 carriers (ε2/ε4, ε3/ε4, ε4/ε4)	Altered response to diet and exercise; higher LDL-C and total cholesterol	Guide personalized dietary and lifestyle interventions to optimize lipid profile and cardiovascular risk	[101,102,103,104]
**Patients undergoing thrombophilia testing with unclear benefit**	Any genotype with endothelial biomarkers	Conventional thrombophilia tests often low-yield; unnecessary testing can increase cost and anxiety	Use genotyping combined with functional markers to reduce non-informative tests and focus preventive resources	[105,106]

## Data Availability

The data for this manuscript are derived from publicly available published clinical trial and study results from 1975 to 2025.
